# Long-Term Oro-Dental Effects of Chemotherapy in a Pediatric Patient: A Case Study and a Proposed Oral Care Protocol

**DOI:** 10.3390/jcm14020603

**Published:** 2025-01-18

**Authors:** Sasima Puwanun, Rungarun Kriangkrai

**Affiliations:** 1 Department of Preventive Dentistry, Faculty of Dentistry, Naresuan University, Phitsanulok 65000, Thailand; 2 Department of Oral Biology, Faculty of Dentistry, Naresuan University, Phitsanulok 65000, Thailand; rungarunk@nu.ac.th

**Keywords:** oral care protocol, microdontia, chemotherapy, dental disturbance, histopathology, case report

## Abstract

**Background**: Chemotherapy (CMT) in children can disrupt dental development and calcification, causing long-term dental issues, but good dental care and habits can help improve quality of life. This case report examines permanent dental disturbances in a 7-year, 4-month-old girl undergoing CMT, explores the histology of microdontia, and outlines an oral treatment plan for CMT management. **Methods**: Clinical examination revealed microdontia and a groove crossing the cervical area (chronological hypoplasia), which were assessed using panoramic radiographs and histological analysis. The patient was monitored for five years, and microdontia was extracted for orthodontic reasons. A tailored treatment plan was implemented to maintain oral health during CMT. **Results**: Clinical and radiographic findings indicated tooth agenesis, rudimentary form, chronological hypoplasia, and microdontia. Histological analysis showed reduced odontoblast counts, abnormal dentinal tubules, thinner pre-dentin, and interglobular dentin (hypocalcification) surrounded by globular dentin (normal calcification). CMT-related microdontia caused inflammation with dilated blood vessels in the pulp. A high fever during CMT led to a groove in the enamel of all teeth, presenting as chronological hypoplasia. No new dental caries was observed over the follow-up. **Conclusions**: This report highlights long-term dental disturbances from CMT in permanent dentition and associated histopathological changes. It proposes an oral care protocol for managing these issues. Maintaining oral hygiene and preventing caries during the five-year follow-up reduced CMT side effects and improved the quality of the patient’s life.

## 1. Introduction

The global average age at diagnosis for malignant childhood cancer is 10 years. Among children aged 0 to 14, the average age is 6 years, while for adolescents aged 15 to 19, it is 17 years [1]. Within Thailand, the incidence of malignant childhood cancer is greatest in children aged 0–4 years [2]. Leukemia, lymphoma, and brain tumors are the most common malignancies. Astrocytoma is the most common type of brain tumor in children. The 10 year survival rate for low-grade astrocytoma (grade 1 and 2) is more than 90% in high-resource settings [3]. A multiple-treatment approach including both surgery and chemotherapy (CMT) is often used to combat the malignancy. Unfortunately, the side effects of CMT can lead to various dental disturbances in the oral cavity. CMT can interrupt the function or synthesis of nucleic acids in all developing cells. Little et al. reported that the most common complications of CMT in cancer patients younger than 6 years old are dental developmental disturbances such as tooth agenesis, complete or partial arrest of root development, tapered root, early apical closure, globular and conical crowns, dentin and enamel opacity, microdontia, enlarged pulp chamber, taurodontism, and abnormal occlusion [4]. CMT agents can affect the dentition of young patients in various possible stages of dental development and dental calcification. Many studies have reported dental anomalies after the use of multiple CMT agents [5,6,7] in combination. To date, CMT agents have been studied in limited-duration animal models, in vivo, which cannot reveal long-term effects such as anatomical and histological changes in dental structures. Knowledge of the mechanisms of CMT’s effects on the development of permanent dentition is still limited.

Dental infections can increase complications in oncological treatments. The risk of oral infection can be reduced through initial evaluation, treatment planning, and preventive measures. It is vital to talk with parents or caretakers about dental care in connection with CMT, the danger of systemic complications associated with poor daily habits, and the benefits to the child’s quality of life that good daily habits can bring. Thus, a comprehensive dental treatment protocol beginning with dental treatment before the start of CMT continuing through the CMT maintenance phase is very important. Radhika et al. (2019) reported that patients who received an oral care protocol from nurses (intervention group) were able to slightly delay the oral complications during their stay in the oncology ward [8]. Various institutions have established oral care protocols for CMT patients to reduce chemotherapy-induced oral mucositis and other oral complications [9,10,11]. The parents/caretaker have the most time available to support oral care, since they are with the patient both in the hospital and at home. Therefore, the oral care protocol should be provided to both parents/caretakers and patients to emphasize the importance of oral health to cancer treatment [12].

The American Academy of Pediatric Dentistry (AAPD) clinical affairs committee introduced best-practice recommendations for pediatric patients receiving chemotherapy in 1986, and these were most recently updated in 2023 [13]. Compliance of the patient and their parents or caretakers is essential to an effective oral care regimen for patients receiving CMT.

The purpose of the case study presented here is to diagnose, treat, and document permanent long-term dental disturbances occurring as side effects from CMT in a pediatric patient, address the limited histological knowledge of microdontia, and develop an oral care protocol throughout CMT treatment.

## 2. Case Presentation

Advance informed consent for participation in this study was requested and received from the patient and her parents for the publishing of all images, clinical data, and any other data included in the manuscript. The operation was conducted in accordance with the Declaration of Helsinki, and the protocol was approved by the Ethics Committee of the Naresuan University Institutional Review Board (IRB No. P1 0111/2566) on 18 September 2022.

In September 2017, a pediatric dental patient, a 7-year and 4-month-old girl, first had contact with the Pediatric Dental Clinic of Naresuan University Dental Hospital in Phitsanulok, Thailand through an outreach program screening students at a local Special Education Center for dental issues. The patient sought treatment for severe caries. Her parents provided her medical history, explaining that the patient had undergone two operations for the removal of an astrocytic tumor. The first operation was in April 2012 (age 1 year, 11 months) and it was followed by CMT. After the operation, the girl lost her vision and never regained it, only being able to sense light. Additionally, the patient has hypothyroidism, also a result of the operation. The second operation, also followed by CMT, was in April 2016 (age 5 years, 11 months).

**Figure 1:**
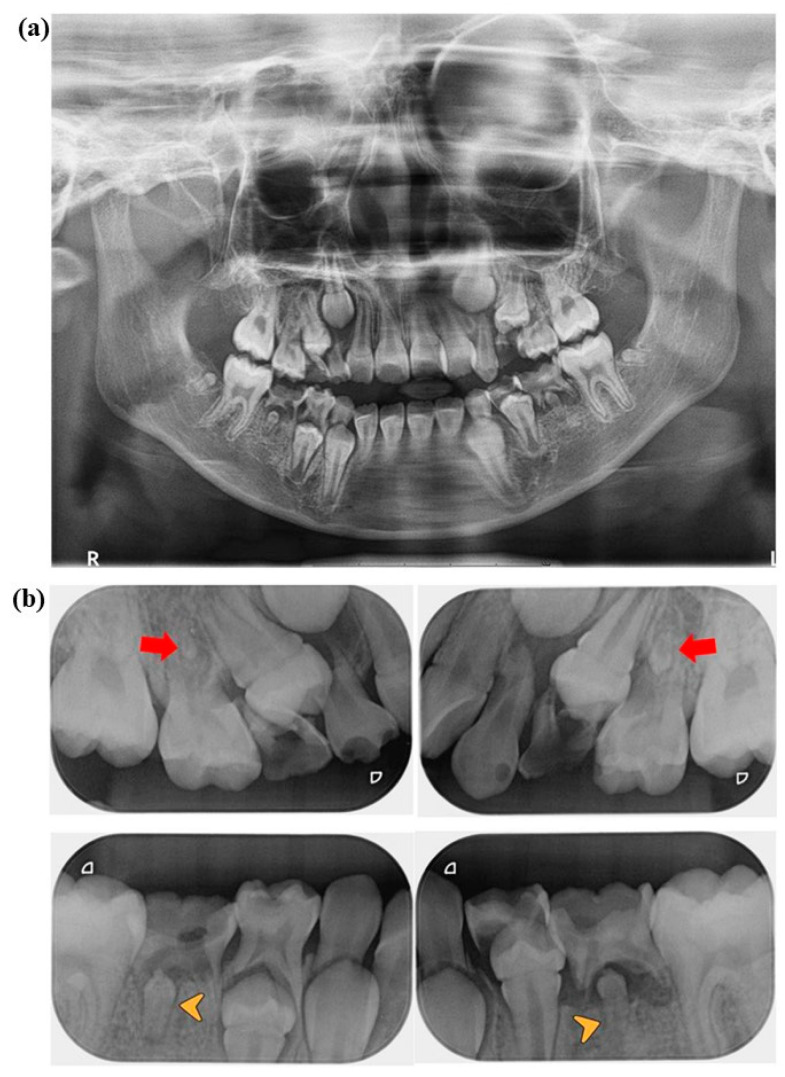
(**a**) Panoramic radiograph and (**b**) periapical radiographs were taken of the 7-year and 4-month-old patient (first visit). The film shows the patient’s primary teeth with a decayed-missing-filled-teeth index (DMFT) of 10. (**b**) The red arrows show rudimentary formations on tooth numbers 15 and 25. The yellow arrow heads show microdontia on tooth numbers 35 and 45.

An intraoral and radiographic examination later at the clinic revealed that the patient was in the period of mixed dentition with permanent incisors and first molar present. Figure 1 shows the radiographic images taken during the first dental visit. The decayed-missing-filled-teeth index (DMFT) on permanent teeth was 0. The primary molars and canine teeth exhibited poor oral hygiene, with a DMFT of 10 on the primary teeth, as shown on a standard panoramic radiograph (Figure 1a) and periapical radiographs (Figure 1b). Extensive destruction of teeth and pathological changes in the regions surrounding the tooth roots of tooth numbers 54, 64, 74, 75, and 85 were diagnosed as pulp necrosis with chronic apical periodontitis. Carious lesions in the dentin area of tooth numbers 53, 63, 65 and 84 were diagnosed as dental caries. Additionally, a carious lesion in the enamel area of tooth number 55 was diagnosed as enamel caries. The dental treatment plan involves tooth removal, restoration with composite filling, and a stainless-steel crown under local anesthesia, which are all invasive (bleeding) procedures.

The oral care protocol for CMT patients (see Section 3) designed in the current study is based on clinical experience. Parents/caretakers and the patient should meet in the treatment room and speak with them about the possible short-term and long-term side effects of CMT on the oral cavity (see also Section 3.1, number 4). Try to raise awareness of the benefits of good oral hygiene to reduce the risk of complications with systemic disease and to increase quality of life. When evaluating the patient during the meeting, praise good dental care that is observed and raise concern about poor dental care that is observed, with the goal of positively motivating the parents/caretakers and patient to follow the recommended personalized oral care protocol. At the same meeting with the parents/caretakers and patient, talk about the basics of good oral hygiene (see also Section 3.1, number 5), for example, using a soft nylon brush without causing bleeding across a wide range of platelet counts, along with fluoridated toothpaste and flossing without traumatizing gingival tissue, as well as diet. The instruction is to instill the knowledge and skills needed for effective routine oral care to prevent common dental issues such as dental caries, gum disease, and mucositis (the most common oral manifestation in chemotherapy patients). Dental scaling and cleaning were performed, and parents/caretakers were taught/reminded about good oral hygiene practices for the management of the enamel caries in tooth number 55. The decayed tooth numbers 53, 63, and 84 were restored with tooth-colored materials (Filtek^TM^, 3M^TM^, Saint Paul, MN, USA) and a stainless-steel crown (ESPE™ Stainless Steel Primary Molar Crowns, 3M™, Saint Paul, MN, USA) in the case of the extensively decayed cavity in tooth number 65. Tooth numbers 54, 64, 74, 75, and 85 with poor prognosis due to pulpal infection and non-restorability were extracted and proper wound closure was ensured. A fixed space maintainer (lingual holding arch), composed of a hard round spring wire (Leowire^®^, Leone^®^, Florence, Italy) and dental bands (Victory Series™ First Molar Bands, 3M™, Saint Paul, MN, USA), was placed on tooth numbers 36 and 46 in the lower arch of the current patient to prevent mesial shift of the lower first permanent teeth. Oral hygiene is monitored every 3 months as per the prevention oral health planning (see also Section 3.2, number 4). Preventive oral health planning). The prevention plan was performed in the dental clinic, for example: dental scaling, professional dental fluoride treatments (fluoride used in dental clinics), sealants, and monitoring of oral conditions [13].

The patient was scheduled for the third round of CMT without any operation in Feb 2019 (age 8 years and 9 months). In this and both the previous rounds, the sole CMT agent was an IV vinblastine dose of 6 mg/m^2^ once per week for 70 weeks. During-CMT, the patient’s absolute neutrophil count (ANC) ranged from 1510 to 3161 per microliter, and the platelet count ranged from 270,000 to 645,000 platelets per microliter. From ANC, if it was below 2000 per microliter (mm^3^), invasive dental treatments were postponed until the ANC was above 2000 per mm^3^, according to the oral care protocol (see also Section 3.2, number 3). In this case, the patient did not require emergency treatment at that time. Therefore, she did not need antibiotic prophylaxis before treatment.

Oral hygiene instructions, scaling, and fluoride application were provided as non-invasive procedures every 3 months, with no new dental caries. Following the protocol, the patient did not need antibiotic prophylaxis before treatments.

**Figure 2:**
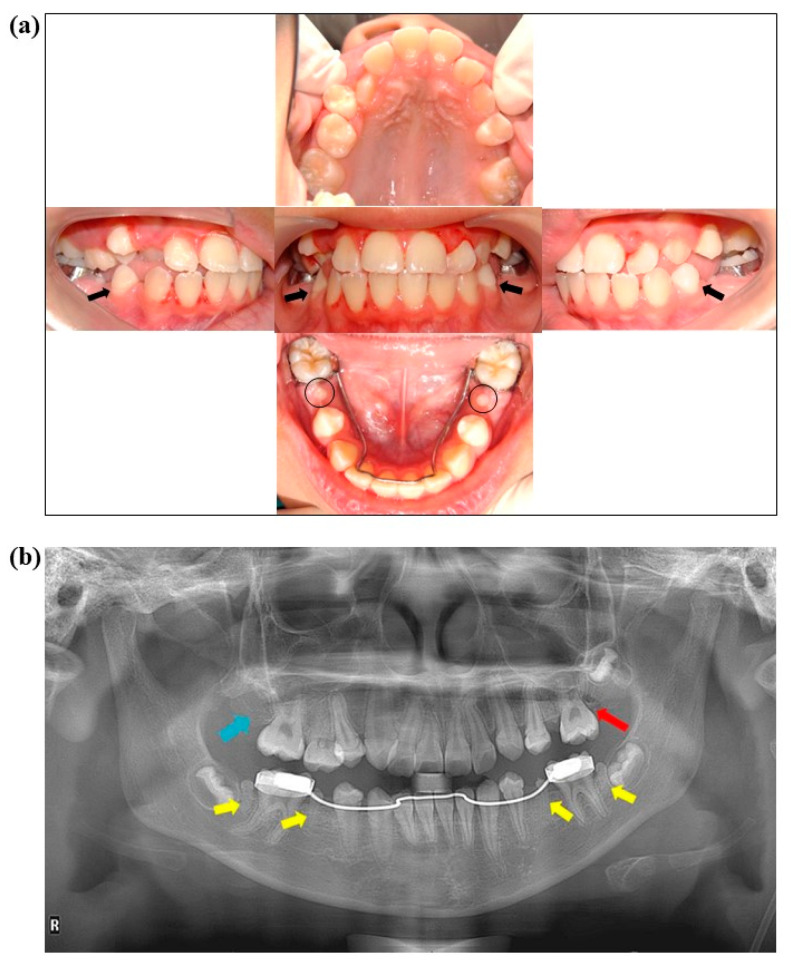
These images were taken of the patient at age 12, five years after completing dental treatment. (**a**) Intraoral pictures show microdontia on tooth numbers 35 and 45 (black circles). Black arrows show the cervical line on the enamel of tooth numbers 34 and 44 (chronological hypoplasia). A lingual holding arch was placed on the lower arch to prevent mesial shift of tooth numbers 36 and 46. (**b**) This panoramic radiograph was taken after the completion of dental treatment. It shows a DMFT of 0 on permanent teeth. The yellow arrows show microdontia on tooth numbers 35, 37, 45, and 47. The blue arrow shows where tooth number 17 is missing, and the red arrow shows a minor calcification mass on tooth number 27.

In May 2022, at the age of 12 years, the patient returned to the clinic for a dental evaluation before orthodontic treatment. Examination of intraoral (Figure 2a) and radiograph pictures (Figure 2b) revealed microdontia in tooth numbers 35, 37, 45, and 47 (Figure 2b, yellow arrows). It was also observed that tooth numbers 17 and 27 were missing (Figure 2b, blue and red arrows, respectively). Cervical lines on enamel (chronological hypoplasia) were found in all first permanent premolars (Figure 2a, black arrows). The overall oral hygiene could be rated as “fair” due to generalized gingivitis associated with the presence of biofilm with no carious lesions (Figure 2a). The orthodontist recommended removing the microdontic teeth, since they were non-functional, to facilitate the alignment of the other teeth.

## 3. Oral Care Protocol for CMT Patients

**Figure 3:**
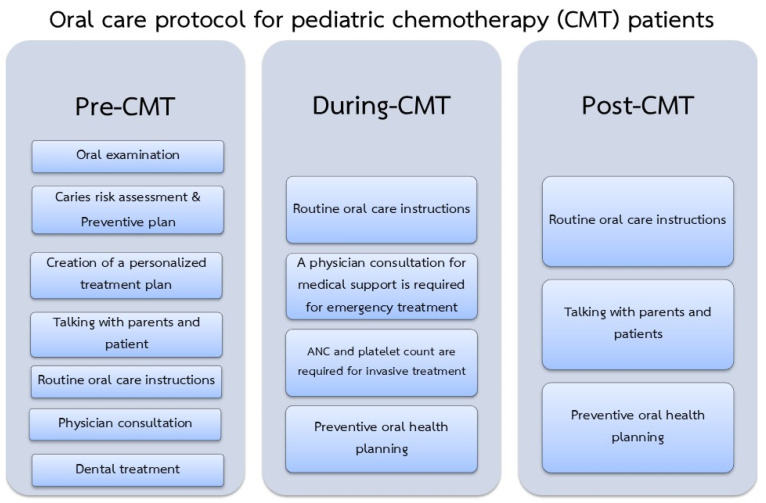
Proposed oral care protocol form before, during, after CMT.

The oral care protocol for CMT patients can be divided into 3 periods: Pre-CMT, During-CMT, and Post-CMT (Figure 3).

Each of the three protocol periods is outlined in more detail below.

### 3.1. Pre-CMT Period

1. Oral Examination Including Radiographic Examination
2. Caries Risk Assessment and Prevention Plan
3. Creation of a Personalized Treatment Plan Based on the Individual Patient’s Situation
4. Discussion with Parents/Caretakers and Patient About Dental Care
5. Routine Oral Care Instructions (For Home Use)

The routine oral care instructions consisted of tooth brushing, 0.2% chlorhexidine mouth rinse (following the dentist prescription), and 0.9% saline rinse [9].

6. Physician Consultation

Upon receiving the patient’s medical history and speaking with her mother, the patient’s oncologist was consulted to ask if the patient’s condition allowed her to receive the prescribed dental treatment. This is a vital step, since the oncologist’s knowledge of the case can help the dentist plan dental treatment in the safest manner and schedule the treatment so that there is no medical interference to or from the CMT.

7. Dental Treatment [13]

- All dental treatment should be completed before beginning CMT. If that is impossible, temporary restorations can be placed, and non-acute dental treatment can be delayed until the patient’s hematological status is stable (see also Section 3.2, number 3).
- Plan treatments by prioritizing symptomatic caries, lesions, or those at risk of irreversible pulpitis, removing infections, eliminating sources of soft tissue irritation, extracting hopeless teeth, and refilling faulty restorations. Trauma from dental procedures such as tooth removal or restoration with a stainless-steel crown could damage the oral mucosa or alveolar bone (the bone that supports the tooth), increasing the risk of bleeding and infection.
- Endodontic treatment in primary teeth should be evaluated and treated before initiating CMT. Some studies suggest that caries and pulpal lesions should be extracted to prevent furcation infections in primary teeth, which could be life-threatening during CMT. Failure of the pulpal treatment could occur during the immunosuppression period.
- Endodontic treatment in permanent teeth with symptoms should be completed in one visit, and the success of the treatment should be assessed at least one week before initiating CMT. If symptoms are present, treatment may be delayed until the patient’s hematological status is stable (see also Section 3.2, number 3). Optional treatments include pulpectomy or extraction.
- Orthodontic appliances and space maintainers should be evaluated to ensure they do not irritate soft tissue and to maintain good oral hygiene. Orthodontic treatment can start at least 2 years after the completion of cancer therapy and being declared cancer-free.
- Teeth with a poor prognosis for periodontal lesions, such as those with furcation involvement, infection, and mobility, should be extracted.
- Primary teeth with mobility due to natural exfoliation may remain in the oral cavity if the patient is asymptomatic and maintains good oral hygiene (no dental plaque).
- Extractions should be scheduled at least 10 to 14 days before starting CMT. Use atraumatic procedures during the extraction to allow for adequate healing and reduce the risk of systemic complications [4].

### 3.2. During-CMT Period

1. Continue Providing Patient with Routine Oral Care Instructions to Prevent Mucositis, Potential Oral Complications, and Reduce Medical Complications During CMT [4]
2. Discuss Emergency Treatment Plans with the Physician for Medical Support Before Any Dental Procedures. Elective Dental Treatments Should Be Postponed Until the Patient’s Hematological Status Is Stable
3. The Dentist Should Receive the Patient’s ANC and Platelet Count Before Planning the Treatment for Each Visit. It Is Important to Be Aware of the Risk of Bacteremia and Excessive Bleeding in CMT Patients. AAPD Reported That for Pediatric Patients Receiving Immunosuppressive Therapy and/or Head and Neck Radiation, the Standard Regimen Does Not Include Antibiotic Prophylaxis for Noninvasive (Non-Bleeding) Procedures. For Invasive Procedures, the Patient’s ANC and Platelet Count Should Be Evaluated as Follows [13]:

ANC

- More than 2000/mm^3^: No need for antibiotic prophylaxis.
- 1000 to 2000/mm^3^: Clinical judgment depends on the patient’s health status. If infection is present at the site of treatment, the patient’s oncologist should be consulted regarding antibiotic prophylaxis before use.
- Less than 1000/mm^3^: Treatment only in case of emergency under consultation with the oncologist and using antibiotic treatment before the dental procedure.

Platelet count

- More than 60,000/ mm^3^: No antibiotic prophylaxis needed.
- Less than 60,000/mm^3^: Avoid invasive procedures. In case of emergency, the oncologist should be consulted regarding treatment with invasive procedures before proceeding.

4. Preventive Oral Health Planning

Preventive Oral Health Planning was personalized through professional oral care in a clinic to prevent oral health problems. These measures include regular dental check-ups every 3 months. 

### 3.3. Post-CMT Period [13]

1. Continue Providing Routine Oral Care Instructions
2. Continue Communicating with Parents (Caretakers) and Patients About Potential Acute and Long-Term Side Effects of CMT on Dental Development
3. Preventive Oral Health Planning

## 4. Materials and Methods

### 4.1. Collected Teeth

The teeth with microdontia were collected from the patient through maxillofacial surgery (with written informed consent as above) at Naresuan University Dental Hospital. The teeth were fixed with 10% formaldehyde and sent for histological analysis to the Dental Science Research Center at Naresuan University’s Faculty of Dentistry. To conduct a comparative study, the two microdontia teeth and an exfoliate primary tooth with a non-contributory medical history from a different patient were all prepared for histological processing and then stained using identical procedures.

### 4.2. Histology Fixation, Staining, and Examination

After fixation, the teeth were washed in phosphate-buffered solution (PBS) and decalcified in a 10% ethylene diamine tetra-acetic disodium (EDTA-2Na) solution with a pH of pH 7.6. The samples were dehydrated through a graded series of ethanol solutions and then embedded in paraffin. Next, 4–5 μm-thick paraffin-embedded serial longitudinal sections were prepared. In order to better show discrete dental structures, the specimens were counterstained with hematoxylin and eosin (H&E) (C.V. Laboratories Co., Ltd., Bangkok, Thailand) and Masson’s trichrome protocols (Sigma-Aldrich, Steinheim, Germany). Microscopic images were taken using an Olympus BX-60 microscope (Olympus Corporation, Tokyo, Japan) and dedicated Cell Sens software (OLYMPUS cellSens Dimension 2.3).

### 4.3. Counting Cells and Measuring Pre-Dentin Layer

The number of odontoblast cells in the normal control tooth and the microdontia were counted from the histopathological images at a magnification of ×25.2. Each image was randomly overlaid with a rectangular grid (450 µm wide and 283 µm high) with six areas per tooth. The odontoblastic cell numbers are presented as the average cells/area.

The thickness of the pre-dentin layer in a normal control tooth and the microdontia was measured. Each image was randomly overlaid with a rectangular grid (450 µm wide and 283 µm high), with six areas per tooth. The thickness of pre-dentin layers is presented as the average thickness [14].

## 5. Results

**Figure 4:**
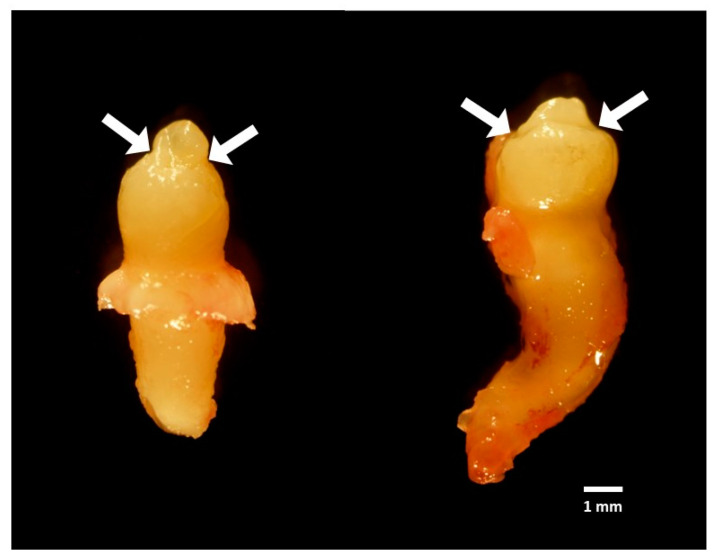
The gross anatomy of microdontia on tooth numbers 45 and 47 (tooth height < 50% of expected size). White arrows showed the line on enamel (chronological hypoplasia). Scale bar = 1 mm.

The patient’s premolar and second molar teeth were adversely affected by vinblastine therapy, resulting in microdontia in tooth numbers 35, 45, 37, and 47, as well as rudimentary formation in tooth numbers 15 and 25. Tooth numbers 17 and 27 never formed. However, a minor calcification mass was discovered to have formed at area 27 (Figure 2b: red arrow). As per the treatment plan, the right premolar and second molar teeth (microdontia teeth 45 and 47) were extracted. The gross anatomy of the microdontia exhibited a well-defined shape with fully formed root (Figure 4) The height of the microdontia was less than 50% of the average height of the normal teeth in the control group (microdontia height = 10.09 mm, average control tooth height = 22.5 mm) [15]. Tooth numbers 45 and 47 were subsequently processed for histological analysis.

**Figure 5:**
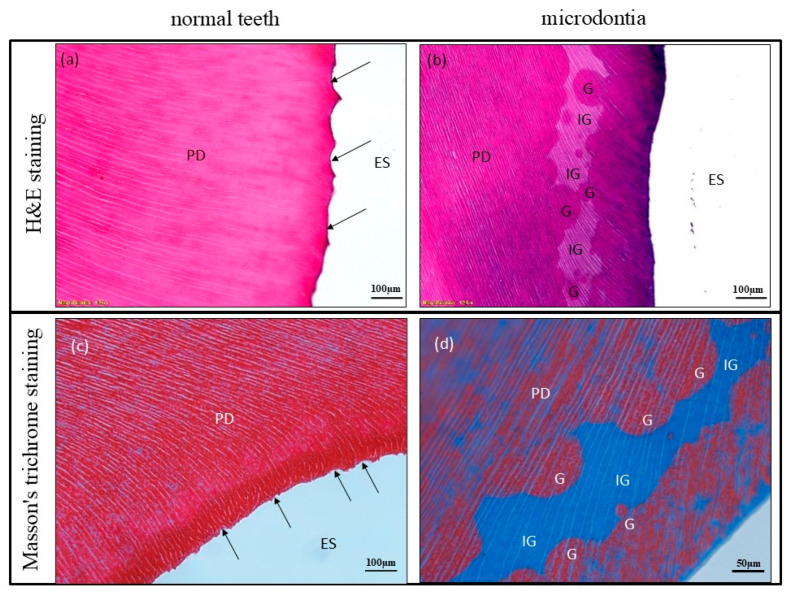
(**a**,**c**) A decalcified section of a normal tooth (control) shows normal primary dentin lining near the scalloped dentinoenamel junction (DEJ: black arrows), (**a**) was stained with H&E, (**c**) was stained with Masson’s trichrome. Scale bar = 100 μm. (**b**) Microdontia shows decreased mineralization in primary dentin, indicating a large region of interglobular dentin (IG: pale pink area), surrounded by globular dentin (G: dark pink area) (H&E; Scale bar = 100 μm). (**d**) Microdontia shows decreased mineralization in primary dentin indicating a large region of interglobular dentin (blue area), surrounded by globular dentin (red area) (Masson’s trichrome staining; Scale bar = 50 μm). ES = Enamel space, G = globular dentin, IG = interglobular dentin, PD = primary dentin.

Histological analysis showed that the decalcification process degraded the enamel tissue. As a result, the enamel is not visible in the images and is instead represented as enamel space (ES). When compared to normal teeth in the control (Figure 5a,c: tooth number 14), the primary dentin of the microdontia showed evidence of an incomplete mineralization process, which likely began before tooth eruption (Figure 5b: tooth number 47). A significant area of interglobular dentin (IG) was observed in the hypomineralized primary dentin of the affected teeth, suggesting an expanded hypomineralization zone between areas of globular dentin (G) (Figure 5b). Masson’s trichrome staining revealed positive blue staining in the low mineralization area (Figure 5d: tooth number 47), whereas the control tooth showed red staining in the primary dentin, indicating a typical mineralization pattern (Figure 5c).

**Figure 6:**
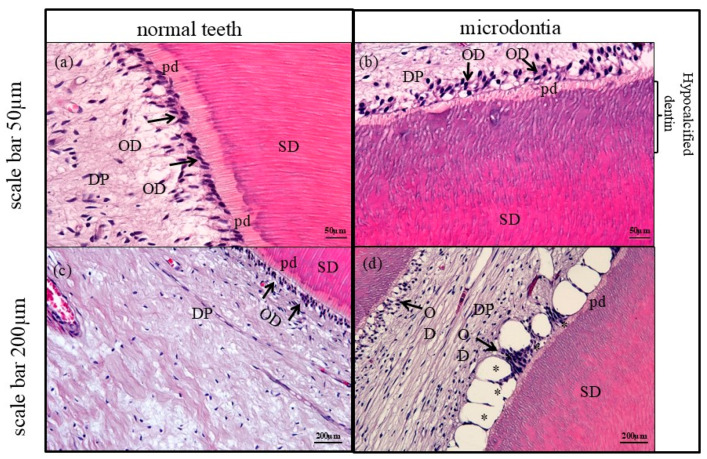
(**a**,**c**) A normal tooth (control) shows normal secondary dentin (SD) lining nearby pulpal tissue (DP) with normal pre-dentin (pd) (newly formed dentin before mineralization) secreted by healthy odontoblasts (OD: arrow), H&E. (**a**) has a scale bar = 50 μm, (**c**) has a scale bar = 200 μm. (**b**) Microdontia tooth displayed a decrease in the odontoblast cells (OD: arrow) lining with irregularity of dentinal tubules with thinning of pre-dentin (pd) product, compared to those found in the control. Additionally, mature dentin bound by pre-dentin showed lower calcification product than those of normal dentin, H&E. Scale bar = 50 μm. (**d**) Vinblastine chemotherapy induced disorganized odontoblast cells and dental pulp edema (asterisk) in microdontia, H&E. Scale bar = 200 μm.

**Figure 7:**
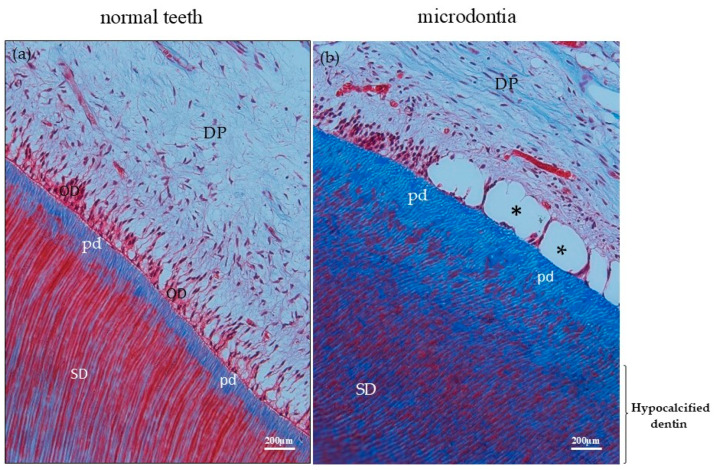
(**a**) Decalcified section of a normal tooth (control) shows normal secondary dentin lining. (**b**) Mature dentin bound by pre-dentin shows less calcification product than normal dentin as well as a lower number of odontoblast cells, and dental edema (asterisk). Masson’s trichrome staining. Scale bar = 200 μm. DP = dental pulp, SD = secondary dentin, pd = pre-dentin, asterisk = dental pulp edema.

In comparison to the normal control teeth (Figure 6a: tooth number 14), the microdontia showed a decrease in odontoblastic cells (Figure 6b: tooth number 47). The average number of odontoblastic cells per area in microdontia was 59.83 cells/area, compared to 93.83 cells/area in the normal control tooth. The thickness of pre-dentin, which is the newly produced layer of dentin matrix before mineralization, was thinner in the microdontia compared to the normal control tooth. The average thickness of pre-dentin in the microdontia was 11.88 µm, compared to 36.95 µm in the normal control tooth. In the microdontia, there was an irregular lining of the dentinal tubules, while the normal control tooth showed a smooth lining of the dentinal tubules. A portion of these odontoblast cells displayed vinblastine chemotherapy-induced dental pulpal edema (Figure 6d and Figure 7b: asterisk: tooth number 47), compared to the normal control teeth (Figure 6c: tooth number 14). This is consistent with a study by Satoh et al., in which edema of the dental pulp was found in mice treated with antitumor drugs in vivo [16]. Compared to normal dentin (Figure 7a: tooth number 14), the mature dentin in microdontia, bordered by pre-dentin in the secondary dentin, showed a reduced calcification product (Figure 7b: tooth number 47).

## 6. Discussion

The focus of this study is to suggest a protocol that incorporates both the patient’s and their parents’ or caretakers’ awareness of oral hygiene. Dentists should discuss with the patient and their parents or caretakers about the importance of prioritizing preventive oral care and regular dental visits to prevent possible complications from CMT. In this case, the patient was assessed for caries risk, and a prevention plan was created. Her mother was told about the advantages of maintaining good oral hygiene. A later visit by the patient showed how much her oral hygiene had improved, with no tooth decay or mucositis, which is a most common lesion in chemotherapy patients.

CMT agents affect the number and growth of cells involved in dental development and cause disturbances in tooth development and eruption. There is no precise information on how permanent teeth develop after birth in individuals who have undergone CMT. The exact nature and extent of damage depends on the age of patient at the beginning of CMT and the half-life of the drug regimen [17]. This report will compare this patient’s atypical dental development with the normal stages of dental development [6]. The lack of specificity of the CMT agent in differentiating tumor cells from active normal cells, such as odontoblasts and ameloblasts, can have negative effects. As a result, abnormalities of tooth developments can occur, including incomplete calcification, enamel hypoplasia, agenesis, microdontia [18].

**Figure 8:**
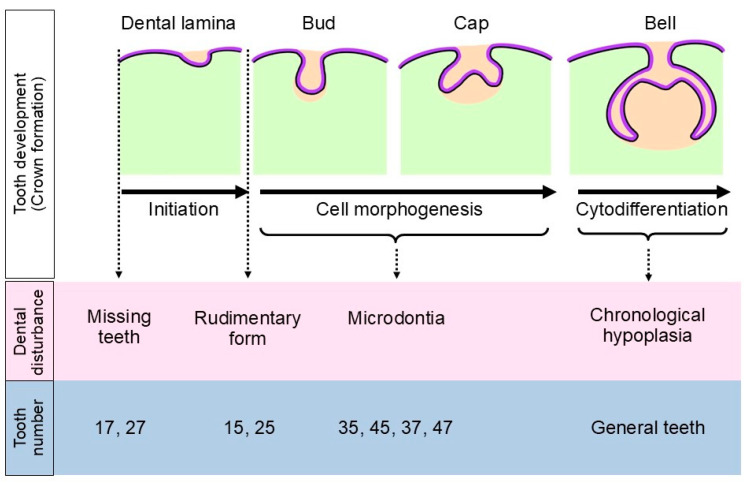
Summary of dental disturbances related to tooth development (crown formation) with tooth numbers from this case.

In this case, the patient began CMT treatment at 1 year and 11 months of age. She received only vinblastine for all CMT sessions. During the clinical examination, microdontia was found in the lower second premolars and lower second permanent molar teeth. CMT can disturb cell morphogenesis during any stage of tooth formation, from the bud stage to the cap stage to the early bell stage before calcification of the tooth germ (Figure 8). The mineralization of lower second premolars and lower second permanent molar teeth begins at 1.25–2.5 and 2.5–3 years of age, respectively [19]. The size and shape of this patient’s teeth are consistent with the findings of a study by Gören et al., that the most prevalent dental anomalies after chemotherapy (in children younger than 4 years of age) are found in premolars and second molars. Other teeth, such as incisors, canines, and first molars begin to mineralize shortly after birth [20].

The patient’s upper second premolar teeth developed in rudimentary form, meaning they were not fully developed and were smaller in size compared to microdontia. The first CMT may disrupt the initiation stage (before the cell morphogenesis stage) of tooth development (Figure 8). The mineralization of these teeth typically begins at 2–2.5 years of age.

Missing upper second permanent molars were found in the patient’s orthopantomogram. CMT likely disrupted the tooth development process before the initiation stage and thereby impaired the development of the tooth germs (Figure 8). Another factor is that her hypothyroidism may have affected the growth of the upper jaw (maxilla). An insufficient area of the maxilla could obstruct the backward extension of the dental lamina, preventing the formation of the second permanent molar tooth germ. Nanci reported that the formation of permanent molar teeth begins at 20 weeks of pregnancy (for the first permanent molar) and continues to 5 years of age (for the third permanent molar). A growth disturbance at any point during this period may result in missing teeth [21]. The missing upper right third molar (tooth number 18) was excluded from this study. Koussoulakou et al., 2009, reported that the absence of the third molars is an evolutionary adaptation of the human dentition rather than a tooth disturbance. The third molars are at a position in which the morphogenetic field is weak [22]. Agenesis of third molars is typically excluded from studies due to the high prevalence of their absence [23,24,25].

A distinctive band of enamel structures on the buccal surface of the patient’s first premolars (Figure 2a: black arrows) is likely related to high fever that she experienced after the first cycle of CMT. This high fever can be referred to as febrile neutropenia [26], and it is the most common complication following CMT [27]. Ameloblastic cells are sensitive to environmental changes, and they can die. In this patient, enamel formation was disrupted in all teeth, including those with microdontia. The exact effects depend on the timing and duration of the high fever, a situation which is called chronological hypoplasia. After recovery from the high fever, it is possible for enamel formation to return to normal [28,29,30]. In this case, the high fever affected the late bell stage or the cytodifferentiation stage (Figure 8). Chronological hypoplasia can increase the risk of dental caries with or without affecting esthetics. Therefore, dental management, including careful discussions with parents/caretakers about routine oral care and adherence to the “oral care protocol” outlined above, is very important for increasing quality of life for patients.

This patient’s dental histology revealed a decrease in odontoblast cell numbers and improper function of these cells, which altered the characteristics of the pre-dentin and hypocalcified dentin. Several studies have reported related effects. Satoh and colleagues found that vinblastine inhibited the microtubulin spindle in the mitotic cell division phase, leading to fewer odontoblast cells [16]. Consistent with this, Goho C. reported that CMT damages odontoblasts during the actively proliferating phase (M phase: DNA synthesis and replication). However, cells in the nonproliferating phase (germinal phase) are unaffected [31]. Akita et al. and Sasaki and Garant reported that vinblastine alters cell shape and the intercellular transport system on the cytoplasmic microtubules of odontoblasts, leading to decrease pre-dentin production and changes in their properties [32,33]. Miake Y and colleagues reported an irregular dentin that exhibited hypomineralization in the “IG” area and hypermineralization in the “G” area, in vivo [34]. In the hypermineralization area, there was a lower density of collagen fibers and the thick needle shape of calcium deposition which were similar to those in normal dentin. In the hypormineralization area, there was a high density of collagen fibers and smaller calcium deposits. This is consistent with the results of the current study, in which both interglobular and globular areas were observed in the microdontia sections. The dental pulp edema observed may be due to inflammation, since there were inflammatory cells [16] and dilated blood vessels filled with blood cells [35]. Epstein and Miaskowski reported that CMT patients can develop an inflammatory process in the oral cavity [36]. For example, vasodilation increases the permeability of blood vessels, allowing inflammatory cells to move from the bloodstream to the interstitial fluid [37]. Pohl and colleagues reported that a characteristic feature of acute inflammation in the dental pulp is significant vasodilation in the plexus of sub-odontoblastic capillaries. This vasodilation serves to transport immune cells to the affected sites [38].

## 7. Conclusions

Oral care for CMT patients ideally involves a plan tailored from the proposed oral care protocol to the specific needs and circumstances of the patient. Parents, caretakers, and patients should prioritize good oral hygiene throughout the cancer treatment. The effects of dental disturbances are related to the age at which CMT begins and the stage of dental development. The histological examinations of the CMT patient in this study and the normal control patients showed differences, including a lower number of odontoblast cells, disorganization of odontoblast cells and dentinal tubule lining, thin pre-dentin, and dental pulp edema. This case report and proposed protocol can help dentists create treatment plans for managing CMT and its long-term effects on dental health. Preventive oral health planning could prevent dental caries, and maintaining good oral hygiene over five years of follow-up can reduce CMT side effects and improve the quality of the patient’s life. 

## Data Availability

No new data were created or analyzed in this study. Data sharing is not applicable to this study.
